# Metabolic inflexibility in women with PCOS is similar to women with type 2 diabetes

**DOI:** 10.1186/s12986-018-0312-9

**Published:** 2018-10-20

**Authors:** Nicholas T. Broskey, Charmaine S. Tam, Elizabeth F. Sutton, Abby D. Altazan, Jeffrey H. Burton, Eric Ravussin, Leanne M Redman

**Affiliations:** 1 Pennington Biomedical Research Center, 6400 Perkins Rd, Baton Rouge, LA 70808 USA; 2 School of Life and Environmental Sciences and Centre of Translational Data Science, University of Sydney, Sydney, NSW Australia

**Keywords:** Polycystic ovary syndrome, Metabolic flexibility, Substrate oxidation, Insulin resistance, Hyperinsulinemic euglycemic clamp

## Abstract

**Background:**

An ability to switch between primarily oxidizing fat in the fasted state to carbohydrate in the fed state, termed metabolic flexibility, is associated with insulin sensitivity. Metabolic flexibility has been explored previously in women with polycystic ovary syndrome (PCOS), yet the independent or synergistic contributions of androgen excess and/or insulin resistance is not yet known. Therefore, the purpose of this article was to characterize metabolic flexibility in women with PCOS compared to women of normal BMI, obesity, or type 2 diabetes (T2DM).

**Methods:**

Eighty-six weight-stable women; thirty with either PCOS (*n* = 30), or fifty-six with obesity (*n* = 12), T2DM (*n* = 27), or normal BMI (*n* = 17) underwent a hyperinsulinemic euglycemic clamp and indirect calorimetry to measure insulin sensitivity and substrate oxidation via indirect calorimetry, respectively.

**Results:**

All analyses were adjusted for differences in age, ethnicity, and BMI between groups. Women with PCOS were less metabolically flexible compared to healthy women with obesity (*p* < 0.0001), normal BMI (*p* < 0.0001), but after controlling for glucose disposal rate, were similar to women with T2DM (*p* = 0.99). When dividing women with PCOS above and below the mean cutoff for insulin resistance, the insulin resistant women with PCOS had lower rates of non-oxidative glucose metabolism (*p* = 0.0001), higher levels of percent free testosterone (*p* = 0.04), a higher free androgen index (*p* = 0.006), more visceral adipose tissue (*p* = 0.02), and were less metabolically flexible (*p* = 0.007).

**Conclusions:**

Women with T2DM were as metabolically inflexible as women with PCOS. When stratifying women with PCOS into those who are metabolically flexible and inflexible, the women who are inflexible display greater amounts of visceral fat and androgen excess. The inability to alter substrate use given the physiological stimulus may lead to subsequent increases in adiposity in women with PCOS thereby further worsening the insulin resistance.

**Trial registration number:**

Clinical Trials.gov, NCT01482286. Registered 30 November 2011.

## Background

Polycystic ovary syndrome (PCOS) is the most common endocrine disorder in reproductive aged women with reports suggesting that it affects one in every five to one in 20 women worldwide [1]. Insulin resistance is a prominent characteristic of the disorder occurring in approximately 75% of cases [2]. Insulin resistance and the resulting hyperinsulinemia are proposed to be the underlying deleterious causes for the relationship of metabolic disturbances and reproductive dysfunction in PCOS [3]. The metabolic phenotype of PCOS is exacerbated by increased adiposity, and the prevalence of PCOS is greater with overweight and obesity [4, 5]. Interestingly, compared to women with regular cycles (matched for age and BMI), insulin resistance in women with PCOS is worse, suggesting an influential role for androgen excess in the insulin resistant phenotype [6].

A hallmark of insulin sensitivity is metabolic flexibility, the ability to alter substrate use in response to a physiological stimulus. The switch from primarily oxidizing lipids in the fasted (basal) state to carbohydrates in the fed (insulin-stimulated) state renders an individual as being metabolically flexible and represents a normal metabolic response in individuals considered insulin sensitive [7]. In comparison to individuals who are insulin sensitive, individuals who are insulin resistant are metabolically inflexible and thereby lack the capacity to maximally switch between energy substrates which is thought to lead to the development of insulin resistance and subsequently type2 diabetes [8–10]. Metabolic flexibility has been reported in two studies of adult women with PCOS; however, the findings are conflicting. In comparison to women with normal menstrual cycles, women with PCOS have been shown to have a similar degree of metabolic flexibility [11] or to be less metabolically flexible [12]. However, neither of these two reports are compelling because the underlying pathology of insulin resistance between women with PCOS and controls matched for age, adiposity (BMI), and varying degrees of insulin sensitivity is likely different. In order to understand if the metabolic flexibility is influenced by the unique hormonal phenotype, namely hyperandrogenemia in women with PCOS, metabolic flexibility should be studied independently of insulin resistance, and thereby an appropriate comparison group is women with type 2 diabetes mellitus (T2DM) [13]. To our knowledge, no studies have attempted to disentangle the role of the disordered, metabolic and hormonal phenotype of PCOS with metabolic flexibility. The purpose of this cross-sectional study was to assess metabolic flexibility in women with PCOS in comparison to women with regular menstrual cycles and normal BMI, obesity, or T2DM. We hypothesized that metabolic flexibility in women with PCOS will be attenuated in comparison to women with a normal BMI and women with obesity with normal menstrual cycles, and, given that androgen excess is unique to PCOS, metabolic flexibility will be more blunted than for women with T2DM.

## Methods

This report includes 86 women who completed testing at Pennington Biomedical Research Center in Baton Rouge, Louisiana. Women with PCOS (*N* = 30) were enrolled in the “Effect of weight and insulin resistance on reproductive function in PCOS (PULSE)” study (NCT01482286), which was approved and monitored by the Institutional Review Board at Pennington Biomedical. Data from the comparison groups, T2DM ( *n* = 27), normal BMI (*n* = 17), and obese (*n* = 12) were derived from the Pennington Center Longitudinal Study (NCT00959270) which is a de-identified database of more than 25,000 individuals who have completed phenotyping at Pennington Biomedical since 1992. Clinical endpoints were collected in accordance with Pennington Biomedical standard operating procedures with robust quality assurance audits. All Pennington Center Longitudinal Study procedures and the data analysis plan for this study were approved by the Pennington Biomedical Institutional Review Board. All subjects provided written informed consent prior to initiation of study procedures.

### PCOS participants

Women with overweight or obesity (BMI ≥ 25.0 kg/m^2^) aged 20 to 40 with self-reported history of oligomenorrhea or PCOS were recruited to participate. PCOS was defined by confirmed oligomenorrhea, anovulation, and clinical/biochemical signs of hyperandrogenemia according to the NIH/Rotterdam criteria [14]. PCOS was confirmed during two clinic visits performed approximately 7 days apart and encompassed positive indication of oligomenorrhea (fewer than 8 regular cycles in the past year), clinical and/or biochemical androgen excess (Ferriman-Gallwey hirsutism rating > 8 and/or free androgen index > 3.85 [15]) and anovulation (serum progesterone < 0.3 ng/mL). Potential participants were excluded for other potential causes of androgen excess and medication use with known effects on weight control, glucose intolerance, thyroid production, or antipsychotic medications.

### Comparison group participants

A data query to an electronic database from the Pennington Center Longitudinal Study was used to identify women aged older than 18 years with a completed euglycemic hyperinsulinemic clamp with a single dose of insulin (80 mU/m^2^/min) administered for 120 min. Presence of T2DM was identified from the study eligibility criteria or if subjects self-reported “yes” to diabetes or self-reported “no”, but had a fasting plasma glucose concentration ≥ 126 mg/dL, or for those taking insulin sensitizing medications, a glucose disposal rate of < 5.3 mg/kg/fat-free mass (FFM) + 17.7 as previously defined [16]. Furthermore, women were titrated off of diabetes medications over a 2-week period at baseline prior to testing. Demographic information including age, height and gender were also extracted.

### Anthropometry and body composition

Body weight was measured in the morning after an overnight fast while wearing only underwear and a pre-weighed hospital gown. Dual X-ray absorptiometry (DXA) scans were performed using a General Electric Lunar iDXA whole-body scanner (General Electric, Milwaukee, WI) in the women with PCOS or the Hologic DXA QDR 2000 (Hologic, Marlborough, MA) in the comparison group participants. Percent fat data were all converted to the Hologic QDR 4500A using regression equations determined in a validation study conducted at Pennington Biomedical (unpublished data). Visceral adipose tissue (VAT) was quantified using approximately 40 axial MRI images of 10 mm thickness and at 40 mm intervals across the whole body [17]. SliceOmatic 4.2 image analysis software (Tomovision, Montreal, Canada) was used to analyze images on a PC workstation (Gateway, PIII 500 MHz). All MRI scans were read by the same trained observer.

### Insulin sensitivity

Insulin sensitivity was measured by a single-step hyperinsulinemic euglycemic clamp [18]. The clamp protocol followed the Pennington Biomedical standard operating procedure which requires an intravenous catheter be inserted into an antecubital vein for infusion of glucose and insulin and a second catheter placed retrograde in a dorsal vein of the contralateral hand for blood withdrawal. After three basal blood samples were collected, insulin infusion began with a primed dose followed by a constant infusion (80 mU/min/m^2^) for 120 min. Plasma glucose was clamped at 90 mg/dL in all subjects. Plasma glucose was measured at five minute intervals and exogenous glucose (20% dextrose) was infused at variable rates to maintain plasma glucose concentration. The steady state response to the insulin infusion was evaluated in the last 30 min of the clamp. The mean rate of exogenous glucose infusion during the steady-state period was defined as the glucose disposal rate (GDR). GDR was adjusted for glucose concentrations during this steady-state interval (GDR x average group steady-state glucose/individual steady-state glucose) and also for differences in FFM and metabolic size (FFM + 17.7) [19].

### Substrate oxidation and metabolic flexibility

For 30 minutes during the basal and steady-state periods of the clamp, a ventilated hood and bedside indirect calorimeter (DeltaTrac II metabolic cart, Sensormedics, Yorba Linda, Ca) was used to measure gas exchange and substrate oxidation. Non-oxidative glucose metabolism was calculated as the difference between the GDR (mg/min) and the rate of carbohydrate oxidation (mg/min). Metabolic flexibility was calculated as the difference between the mean respiratory quotient (RQ) in the steady-state period minus the mean RQ in the basal period. Metabolic flexibility was also adjusted for GDR as described previously by dividing metabolic flexibility by GDR [8].

### Clinical chemistry

Glucose and albumin were assayed using the Beckman Coulter DXC 600 Pro (Beckman Coulter Inc., Brea, CA). Immunoassays for sex hormone binding globulin (SHBG), progesterone, and testosterone were assayed using the Siemens Immulite 2000 XPi (Siemens Healthcare Diagnostics Inc., Tarrytown, NY) with chemiluminescent detection at Pennington Biomedical. The calibration range for testosterone is between 20 and 1600 ng/dL with an analytical sensitivity of 15 ng/dL. The calibration range for SHBG is up to 180 nmol/L with an analytical sensitivity of 0.02 nmol/L. The PBRC in-house coefficient of variation for testosterone are between 6 and 8% and 4–6% for SHBG when controls are run (3 and 2 levels, respectively). The University of Virginia Ligand Core completed assays for insulin on the Siemens Immulite 2000 XPi (Siemens Healthcare Diagnostics Inc., Tarrytown, NY). Free Androgen Index (FAI) was calculated by: FAI = [total testosterone × 0.0347 × 100]/SHBG and also using an equation from Vermeulen et al. that includes albumin [20].

### Statistical analyses

All analyses were completed using SAS/STAT® software, Version 9.4 of the SAS System for Windows (Cary, NC, USA). All tests were performed with significance level α = 0.05, and findings were considered significant when p < α. For continuous baseline characteristics and primary and secondary outcomes, group differences were assessed by one-way analysis of variance (ANOVA). To account for the difference in age and race between the groups, age and race were included as a covariate in the ANOVA models. Additionally, to account for the difference in adiposity between the groups, BMI and percent fat mass were also included as a covariate in the ANOVA models separately. Where significant, a Tukey *post-hoc* correction was applied to tests of pairwise differences in least squares means between groups to account for inflation of type I error due to multiple comparisons.

## Results

### Metabolic characteristics of the study groups (Table 1)

Women with PCOS were significantly younger than women with obesity or women who had T2DM (all *p* < 0.0001), but similar in age to women who were normal BMI (*p* = 0.09). Women with PCOS weighed approximately 10 kg more than women with obesity (*p* = 0.005), approximately 2 kg more than women with T2DM (*p* = 0.01), and almost 40 kg more than women with normal BMI (*p* < 0.0001). Fasting glucose in women with PCOS was significantly lower than in women with T2DM (*p* < .0001), but not different from women with normal BMI (*p* = 0.26) or women with obesity (*p* = 0.35). Insulin sensitivity (GDR) normalized for fat-free mass was not different between women with PCOS and obesity (*p* = 0.25) whereas it was significantly lower in women with T2DM (p < .0001) but highest in women with normal BMI as compared to the other three groups (*p* < 0.0001).

**Table 1:** Characteristics of study participants

	Normal BMI (*n* = 17)	Obese (*n* = 12)	T2DM (*n* = 27)	PCOS (*n* = 30)
Age (years)	22.8 ± 3.7^c^	46.1 ± 15.2^b^	58.2 ± 9.9^a^	28.8 ± 4.7^c^
Race (White/Black)	8/9	9/3	24/3	12/18
Weight (kg)	63.8 ± 8.3^c^	86.5 ± 8.8^b^	100.0 ± 10.7^b^	102.6 ± 19.1^a^
BMI (kg/m^2^)	23.7 ± 3.1^c^	33.3 ± 2.5^b^	33.9 ± 3.5^b^	38.9 ± 8.1^a^
Fat mass (%)	27.1 ± 3.5^c^	35.7 ± 3.2^b^	37.6 ± 3.6^b^	45.8 ± 5.7^a^
Fat mass (kg)	17.3 ± 3.6^c^	31.0 ± 5.5^b^	34.4 ± 6.3^b^	47.7 ± 13.4^a^
Fat-free mass (kg)	46.4 ± 5.8^b^	55.5 ± 4.5^a^	56.6 ± 5.6^a^	55.0 ± 8.0^a^
Fasting Glucose (mg/dL)	75.6 ± 9.3^c^	104.5 ± 30.9^b^	136.7 ± 39.3^a^	90.0 ± 6.9^bc^

Absolute values are expressed as mean ± standard deviation. Statistical analyses included age, race, and adiposity as covariates. Means from groups with no shared connecting letters are significantly different from one another

### Substrate oxidation and metabolic flexibility

Substrate oxidation values can be found in Table 2. During the basal (fasting) state, the mean RQ was significantly lower in women with PCOS compared to women with T2DM (*p* < 0.0001), obesity (*p* = 0.0002), and normal BMI (*p* < 0.0001). In response to insulin stimulation, mean steady-state RQ remained lowest in the women with PCOS compared to the other groups although women with a normal BMI had the highest RQ overall (all *p* < 0.0001). Non-oxidative glucose metabolism normalized for fat-free mass was similar between women with PCOS and obesity (*p* = 0.99). However, women with PCOS had higher non-oxidative glucose metabolism values than women with T2DM but lower values than normal BMI controls (both, *p* < 0.0001).

**Table 2:** Substrate oxidation and metabolic flexibility values during a hyperinsulinemic euglycemic clamp

	Basal (Fasted) State				Insulin Stimulated State			
	Normal BMI	Obese	T2DM	PCOS	Normal BMI	Obese	T2DM	PCOS
RQ	0.84 ± 0.03^a^	0.82 ± 0.02^a^	0.83 ± 0.05^a^	0.76 ± 0.04^b^	0.98 ± 0.04^a^	0.95 ± 0.04^a^	0.88 ± 0.05^b^	0.81 ± 0.05^c^
CHO Ox (mg/kgFFM/min)	1.79 ± 0.56^a^	1.47 ± 0.34^a^	1.71 ± 0.88^a^	0.68 ± 0.72^b^	4.51 ± 0.80^a^	3.50 ± 0.76^a^	2.68 ± 0.87^b^	1.60 ± 0.94^c^
CHO Ox (mg/kgFFM + 17.7/min)	1.29 ± 0.38^a^	1.11 ± 0.27^a^	1.30 ± 0.69^a^	0.52 ± 0.54^b^	3.25 ± 0.53^a^	2.65 ± 0.57^b^	2.03 ± 0.68^b^	1.20 ± 0.69^c^
GDR/FFM (mg/kgFFM/min)	–	–	–	–	12.77 ± 3.55^a^	8.69 ± 2.04^b^	4.64 ± 1.14^c^	6.97 ± 3.33^b^
GDR/FFM + 17.7 (mg/kgFFM + 17.7/min)	–	–	–	–	9.22 ± 2.59^a^	6.58 ± 1.56^b^	3.53 ± 0.86^c^	5.19 ± 2.30^b^
NOGM (mg/kgFFM/min)	–	–	–	–	8.26 ± 3.30^a^	5.19 ± 1.77^b^	1.93 ± 1.04^c^	5.37 ± 3.02^b^
NOGM (mg/kgFFM + 17.7/min)	–	–	–	–	5.97 ± 2.42^a^	3.93 ± 1.35^b^	1.46 ± 0.78^c^	3.99 ± 2.14^b^

Absolute values are expressed as mean ± standard deviation. Statistical analyses included age, race, and adiposity as covariates. Means from groups with no shared connecting letters are significantly different from one another. *PCOS:* polycystic ovary syndrome, *T2DM:* type 2 diabetes mellitus, *RQ:* respiratory quotient, *CHO Ox:* carbohydrate oxidation, *GDR:* glucose disposal rate, *FFM:* fat-free mass, *NOGM:* non-oxidative glucose metabolism

**Fig. 1:**
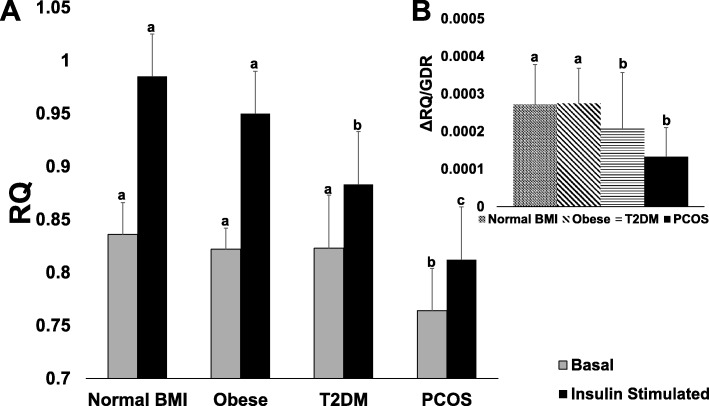
Comparison of metabolic flexibility between women with normal BMI, obesity, type 2 diabetes, and PCOS. Panel **a**: differences in basal and insulin stimulated RQs. Raw unadjusted values are presented but data was adjusted for age, race, and adiposity. Error bars are standard deviation. Differences in basal and insulin stimulated states from groups with no shared connecting letters are significantly different (*p* < 0.05) from one another. Panel **b**: differences in ΔRQ normalized by GDR. RQ = respiratory quotient, ΔRQ:  metabolic flexibility, GDR:  glucose disposal rate, PCOS:  polycystic ovary syndrome, T2DM:  type 2 diabetes mellitus. * = significant *p* < 0.05

Metabolic flexibility (ΔRQ) is shown in Fig. 1. Metabolic flexibility was not different between the women with PCOS and T2DM (0.05 ± 0.03 vs. 0.06 ± 0.04, *p* = 0.98, respectively). However, ΔRQ in women with PCOS was significantly less than women with normal BMI and women with obesity (both *p* < 0.0001). Adjusted [8] ΔRQ was significantly lower in women with PCOS compared to women with normal BMI (*p* = 0.03) and obesity (*p* = 0.06), but similar to T2DM (*p* = 0.99).

### Metabolic and reproductive determinants of metabolic flexibility in women with PCOS

Using a GDR of < 5.3 mg/kg/FFM + 17.7 as previously determined [16], we defined two subgroups (Table 3) of women with PCOS: insulin sensitive (PCOS-IS, *N* = 10) and insulin resistant (PCOS-IR, *N* = 20). As hypothesized, ΔRQ was significantly lower in the PCOS-IR group compared to the PCOS-IS group (0.04 ± 0.02 vs. 0.07 ± 0.04, *p* = 0.007, respectively. Despite similar percentages of total body fat, the PCOS-IR group is ~ 20 kg heavier because of an approximately 27% higher total fat mass. Furthermore, they had twice the amount of visceral adipose tissue than the PCOS-IS group. Hormonal differences showed significantly higher levels of insulin and percent free testosterone and significantly lower levels of SHBG in the PCOS-IR group compared to the PCOS-IS group. No significant differences existed in total testosterone, although the free androgen index was significantly twice as high in PCOS-IR women.

**Table 3:** Comparison of anthropometric, metabolic and reproductive phenotypes between insulin resistant and insulin sensitive women with polycystic ovary syndrome (PCOS)

	PCOS Insulin Sensitive (*N* = 10)	PCOS Insulin Resistant (*N* = 20)	*P*- value
Anthropometric Phenotype			
Age (years)	30.5 ± 5.0	28.0 ± 4.4	0.17
Range, Median	23–29, 31.5	23–37, 26.5	
Weight (kg)	87.9 ± 15.6	110.0 ± 16.5	**0.002**
Range, Median	66.7–109.8, 90.2	87.5–144.4, 109	
BMI (kg/m^2^)	33.8 ± 6.3	41.4 ± 7.7	**0.01**
Range, Median	25.3–44.4, 33.6	32.6–63.9, 38.6	
Fat mass (%)	44.0 ± 6.7	46.6 ± 5.0	0.23
Range, Median	34.2–54.1, 44.9	36.5–58.4, 45.9	
Fat-free mass (kg)	48.5 ± 5.5	58.2 ± 7.1	**0.001**
Range, Median	40.2–55.8, 50.4	47.7–75.6, 57.7	
Fat mass (kg)	39.4 ± 12.2	51.8 ± 12.3	**0.01**
Range, Median	22.8–59.4, 38.5	35.7–77.0, 48.4	
VAT (kg)	1.4 ± 0.79	2.4 ± 1.0	**0.02**
Range, Median	0.6–3.1, 1.2	1.1–4.4, 2.6	
Metabolic Phenotype			
Baseline RQ	0.765 ± 0.05	0.763 ± 0.04	0.93
Range, Median	0.72–0.88, 0.745	0.71–0.83, 0.76	
Insulin Stimulated RQ	0.834 ± 0.06	0.801 ± 0.04	0.08
Range, Median	0.75–0.94, 0.82	0.72–0.85, 0.795	
NOGM (mg/kgFFM/min)	8.79 ± 1.91	3.66 ± 1.72	**< 0.0001**
Range, Median	5.6–11.8, 8.2	0.8–6.6, 4.0	
NOGM (mg/kgFFM+ 17.7/min)	6.39 ± 1.22	2.79 ± 1.30	**0.0001**
Range, Median	4.2–8.4, 6.0	0.6–5.0, 3.0	
Fasting Insulin (uM/L)	10.03 ± 3.47	23.36 ± 9.70	**0.003**
Range, Median	5.7–15.6, 10.3	12.2–53.2, 22.2	
Fasting Glucose (mg/dL)	87.30 ± 5.27	91.35 ± 7.35	0.13
Range, Median	79.0–96.0, 87.5	77.0–105.0, 91.0	
Reproductive Phenotype			
SHBG (nmol/L)^a^	33.71 ± 15.36	24.36 ± 9.52	**0.05**
Range, Median	14.7–67.8, 29.5	9.7–48.5, 23.1	
Total Testosterone (ng/dL)^a^	53.88 ± 17.97	69.22 ± 40.04	0.31
Range, Median	26.5–75, 59.5	22.0–175.5, 62.5	
Free Testosterone (pg/mL)^a^	9.89 ± 3.64	14.91 ± 6.59	0.06
Range, Median	6.4–16.1, 8.6	6.2–27.8, 13.0	
Percent Free Testosterone^a^	1.89 ± 0.48	2.29 ± 0.43	**0.04**
Range, Median	1.1–2.7, 2.0	1.5–3.1, 2.2	
Free Androgen Index^a^	5.71 ± 2.43	9.81 ± 3.51	**0.006**
Range, Median	3.1–9.7, 5.0	4.7–16.1, 10.1	

Data is expressed as mean ± standard deviation. ^a^ = values were averaged over two visits two weeks apart. *VAT:* visceral adipose tissue, *SHBG:* sex hormone-binding globulin, *NOGM:* non-oxidative glucose metabolism. *P*-value font in boldface is statistically significant

## Discussion

The ability of an organism to efficiently alternate between energy substrates in response to physiological stimuli is thought to be characteristic of a healthy metabolism (7). Individuals with insulin resistance lack the ability to fully switch between lipid as a primary fuel source in the fasted state to carbohydrate as a primary fuel source in the insulin-stimulated state [21]. This ability, or inability, to switch between substrates has yet to be adequately explored in women with PCOS. It is not clear whether women with PCOS have the same degree of metabolic inflexibility as T2DM, and moreover, if their metabolic flexibility is explained by their insulin resistant phenotype [11, 12]. This cross-sectional study showed that in comparison to women with normal BMI or obesity and normal menstrual cycles, women with PCOS have a significantly reduced metabolic flexibility which is not different from women with T2DM. However, after taking into account the rate of glucose disposal during the clamp [8] women with PCOS had a more blunted increase in RQ in response to insulin than women with a normal BMI; but similar metabolic flexibility as women with T2DM. Furthermore, within women with PCOS, women that were metabolically inflexible had higher amounts of visceral adiposity and hyperandrogenemia.

Metabolic flexibility is a hallmark of healthy metabolism and is thought to play a substantial role in health and disease [22]. It is important for an individual to be able to switch between oxidizing lipids in the fasted state to carbohydrates in the fed state and back again. The inability to do so has been implicated in the accumulation of ectopic lipid in organs such as the liver and skeletal muscle, and subsequently, development of insulin resistance and T2DM [21]. Metabolic flexibility is often examined as the capacity to metabolize glucose in response to an overload of carbohydrate during a hyperinsulinemic euglycemic clamp. However, Galgani et al. show that the difference in metabolic flexibility during a clamp in individuals with T2DM is the consequence of impaired cellular glucose uptake [8]. After controlling for insulin-stimulated GDR, or the amount of glucose available for oxidation, metabolic flexibility is not different between individuals with and without T2DM. To our knowledge, this is the first study to directly compare women with PCOS to women with T2DM. Not only was the switch in substrate oxidation attenuated in women with PCOS, they also had a substantially lower RQ in both the fasted and the fed states when compared to women with T2DM. It has been previously shown that fat oxidation increases in the fasted state but decreases in the fed state with an increase in adiposity [23]. A pilot study between women with and without PCOS and matched for adiposity has shown that women with PCOS have impaired fat oxidation after an overnight fast and throughout the day, albeit the BMI category was overweight in this study and not obese [24]. This was also shown more recently in women with PCOS and obesity; however, the age of the cohort was much younger than ours [25]. Although both women with PCOS and T2DM were overweight and obese, women with PCOS had a greater percentage of body fat and on average, had class II obesity, which may explain the differences in the RQ in both physiological conditions.

The RQ in both the basal and insulin-stimulated conditions was substantially lower in women with PCOS compared to the women with normal BMI or obesity. However, the metabolic flexibility (ΔRQ) that we observed in women with PCOS was less blunted in response to insulin infusion than another obese PCOS cohort even though the BMI of the women in our cohort [11] was higher. This prior study also reported no differences in metabolic flexibility between women with and without PCOS and concluded that metabolic inflexibility was primarily driven by obesity. However, it is not known whether the metabolic flexibility was independent of GDR. Insulin resistance in addition to the degree of adiposity may be driving metabolic inflexibility. Therefore, we stratified women with PCOS into two categories (insulin resistant or insulin sensitive) to elucidate differences in the degree of insulin sensitivity with these outcomes [16]. The insulin sensitive group was more metabolically flexible, primarily due to an increased metabolic efficiency (higher RQ) in response to insulin infusion. Furthermore, women with PCOS who were insulin sensitive had a lower BMI and significantly less visceral adipose tissue. High amounts of fat in the abdomen as well as ectopic depots have been linked to insulin resistance and metabolic diseases [26] and may impair insulin action in other organs [27]. These data suggest that central adiposity may play a larger role in the etiology of metabolic flexibility in women with PCOS.

The reproductive hormonal milieu may explain metabolic inflexibility in women with PCOS. Di Sarra and colleagues reported an association with hyperandrogenism and a decreased metabolic inflexibility, with free testosterone being a predictor independent of adipose tissue insulin resistance and adiposity [12]. Although we did not observe any significant correlation between androgens and metabolic flexibility in our study (data not shown), we observed increased levels of free testosterone and higher free androgen index in women with PCOS that were insulin resistant compared to women with PCOS that were insulin sensitive. This could simply be due to the increased insulin resistance in the one PCOS cohort and not the other, but we cannot decipher which precedes the other. To that point, testosterone administration in postmenopausal women has been implicated with increased insulin resistance [28]. Furthermore, we reported lower levels of sex hormone-binding globulin (SHBG) in the insulin resistant women with PCOS, which is supported by a recent meta-analysis that suggests SHBG having an inverse relationship with insulin resistance in PCOS compared to women without PCOS [2]. Thus, the increase in circulating free androgens may explain the degree of insulin resistance in women with PCOS. The only significant relationship was a negative association between insulin and metabolic flexibility (*r* = − 0.59, *p* = 0.0009), which supports a previous observation in a PCOS cohort [12]. This may suggest that insulin also contributes to metabolic inflexibility as well in women with PCOS.

Our study is the first to comprehensively characterize metabolic flexibility in women with PCOS against 3 control groups: women with normal menstrual cycles but differing degrees of adiposity or insulin resistance. However, this study is not without limitations. Unfortunately, we were unable to pair-match the groups for BMI or adiposity. In order to disentangle adiposity and insulin resistance from the PCOS phenotype, a wide degree of variability in the control groups was required. Importantly, we adjusted for group differences in both BMI and adiposity in the statistical models and found neither to influence any of the outcomes. The women with T2DM were significantly older than the women with PCOS, but we adjusted for age in all of our statistical models to control for this. The potential post-menopausal status of the women could not be ascertained, which may have an additional effect on the data independently of age. It is important to also note that PCOS may be responsible for up to 30% of T2DM cases in women [29] and therefore the use of T2DM as a comparator group could be subject to bias because we cannot rule out PCOS in the T2DM with clinical criteria. Additionally, we were not able to evaluate the effect of reproductive hormones on metabolic flexibility across all groups because these data were not available in all subjects. Furthermore, we acknowledge that there are other methods including LC-MS that are more robust than our testosterone assay; however, this would be more important if we were not also considering both polycystic ovaries on ultrasound and oligomenorrhea as additional diagnostic criteria. Finally, physical activity level has been implicated as a key determinant of metabolic flexibility [30]. Unfortunately, we do not have any data on physical activity in the groups that were part of the Pennington Longitudinal Study other than they were all classified as sedentary by study design. However, women with PCOS did undergo a VO_2_max test (data not shown) and had an average relative VO_2_max of 19.7 ± 4.2 ml/kg/min with a range from 12.2–31.5 ml/kg/min. Therefore, we are confident that our women with PCOS were sedentary. Furthermore, we found no relationship between VO_2_max and metabolic flexibility (R^2^ = 0.03, *p* = 0.37).

## Conclusions

In conclusion, we show for the first time that after accounting for age, race, and adiposity and controlling for GDR, women with PCOS have similar metabolic flexibility as women with T2DM. Additionally, women with PCOS and insulin resistance typical of individuals with T2DM have more profound hyperandrogenemia, higher visceral fat, and a lower non-oxidative glucose metabolism compared to women with PCOS who are insulin sensitive. This data suggests that intrinsic conditions of PCOS may be mediated through perturbations in glucose transport and/or uptake. The inability to alter substrate use to a given physiological stimulus may lead to subsequent increases in adiposity in women with PCOS with the highest degree of hyperandrogenemia and thereby further worsen the insulin resistance. Future studies are warranted to further investigate the independent and synergistic mechanisms behind these relationships.

## Abbreviations

DXA: Dual X-ray absorptiometry
GDR: glucose disposal rate
PCOS: polcystic ovary syndrome
RQ: respiratory quotient
SHBG: Sex hormone-binding globulin
T2DM: type 2 diabetes mellitus
